# Precision Vaccine Development: Cues From Natural Immunity

**DOI:** 10.3389/fimmu.2021.662218

**Published:** 2022-02-10

**Authors:** Soumik Barman, Dheeraj Soni, Byron Brook, Etsuro Nanishi, David J. Dowling

**Affiliations:** ^1^ Precision Vaccines Program, Division of Infectious Diseases, Boston Children’s Hospital, Boston, MA, United States; ^2^ Department of Pediatrics, Harvard Medical School, Boston, MA, United States

**Keywords:** antigens, natural infection, innate immunity, adaptive immunity, immune system, vaccines, vita-PAMP, adjuvants

## Abstract

Traditional vaccine development against infectious diseases has been guided by the overarching aim to generate efficacious vaccines normally indicated by an antibody and/or cellular response that correlates with protection. However, this approach has been shown to be only a partially effective measure, since vaccine- and pathogen-specific immunity may not perfectly overlap. Thus, some vaccine development strategies, normally focused on targeted generation of both antigen specific antibody and T cell responses, resulting in a long-lived heterogenous and stable pool of memory lymphocytes, may benefit from better mimicking the immune response of a natural infection. However, challenges to achieving this goal remain unattended, due to gaps in our understanding of human immunity and full elucidation of infectious pathogenesis. In this review, we describe recent advances in the development of effective vaccines, focusing on how understanding the differences in the immunizing and non-immunizing immune responses to natural infections and corresponding shifts in immune ontogeny are crucial to inform the next generation of infectious disease vaccines.

## 1 Introduction

Apart from clean drinking water and sanitation, vaccination is one of the most effective medical interventions to avert infectious diseases (1, 2). Despite the great successes of past revolutions in vaccinology, current vaccine technologies may still provide suboptimal protection in vulnerable populations, such as infants (1) and the elderly (3). This is an important unmet need for these vulnerable age-groups, but there are important lessons that can be taken from the breadth of vaccinology work performed for future vaccine design. Moreover, there is a growing need to develop improved vaccine strategies for globally emerging respiratory infections such as tuberculosis, pertussis, influenza and coronaviruses including the pertinent severe acute respiratory syndrome coronavirus 2 (SARS-CoV-2), the causative agent of the severe coronavirus disease 2019 (COVID-19) pandemic.

Historically, the earliest efficacious vaccine was constructed by using live attenuated pathogen small pox to induce protective immunity (4). Subsequently, inactivated, live attenuated, subunit, recombinant, polysaccharide and conjugate vaccines-which all induce humoral and cell mediated immunity-have been used to protect against different viral and bacterial infections. The classical purpose of vaccination is to establish a long-lived state of immunological memory to a given pathogen which can mediate an accelerated response to that pathogen upon secondary infection (5). Vaccines of variable effectiveness against several diseases, including pertussis, influenza and tuberculosis, have been available for many years. In the last few decades, a lack of vaccine induced immunity in distinct populations, especially newborns and older adults, have been reported with impairments attributed to an absence of functional adaptive immunity (6), alternative microbe pathogenicity/activity in newborns/aged compared to adults, and alternative immune regulation processes (7).

Subunit or inactivated vaccines have proven effective against most childhood infections, like tetanus, diphtheria, pertussis, flu and meningitidis (8). The early life window of vulnerability alongside abnormally quick waning immunity from early life vaccinations, elicits major concerns in modern vaccinology (9). A major contributing factor could be the ontological differences in the immune responses. Firstly, upon pathogen challenge or vaccination, the inability of the neonatal and infant immune system to mount a polyfunctional T helper (Th) polarizing response (10), shifting away from protective Th1 and cytotoxic T cell immunity toward dysregulated Th2 and Th17 polarization (11). Secondly, during an infection microbial antigens act as distinct pathogen associated molecular patterns (PAMPs) and viability-associated PAMPs (vita-PAMPs) which elicit broad and long-lasting immune responses. Live vaccines (containing weakened or attenuated form of the microbe) have been able to mimic these responses more closely. Interestingly, growing research suggests that adjuvants and delivery systems may lead to long term immunity by simulating immune responses similar to exposure to live microorganisms (2, 12, 13). Here, we discuss three examples, tuberculosis, pertussis and influenza, in which lessons can be learnt from our current understanding of natural pathogen-specific immunity which can better guide (**Box 1**) novel vaccination strategies to elicit targeted and effective immune activation in neonates and infants, with possible applicability in vulnerable elders and those with immunocompromised status.

Box 1 **Key Considerations for the Study of Natural and Unnatural Immunity to Inform Precision Vaccinology**

Improved understanding of tuberculosis, pertussis and influenza antigen structure, natural immunity and immunopathology may advance the design of novel vaccine candidates. Natural immunity achieved by environmental exposure of microorganisms is responsible for immunological imprinting in humans (14). Yet a number of important caveats need to be addressed, including: i) whether induction of beneficial unnatural immunity can be incorporated into the designing of broad spectrum next generation vaccines [reviewed in (15, 16)], ii) whether these strategies can specifically induce innate immunity and vaccine efficacy in early and later life [reviewed in (6, 11) and (17)], and iii) how can a growing body of vaccine adjuvants, in combination with formulation science of vaccine delivery, such as lipo-nanoparticle based mRNA technologies (3), be used to open up a new toolbox for vaccinologists [reviewed in (12) and (13)].

## 2 *Mycobacterium tuberculosis* and Bacille Calmette Guerin

Tuberculosis (TB) is a leading bacterial cause of mortality worldwide and is caused by an infectious agent named *Mycobacterium tuberculosis* (MTB) (18). Bacille Calmette-Guérin (BCG), the live attenuated *Mycobacterium bovis* vaccine, is the only licensed vaccine for TB to date. It provides protection against disseminated childhood TB but BCG efficacy wanes slowly over time (10-15 years post-vaccination) (19). BCG vaccination has little effect against adult pulmonary TB infection which is the most communicable form of TB (20) and it comparatively carries a greater impact with higher death rates in those over 65 years of age (21). The emergence of drug-resistant strains (22) and limited understanding of protective immunity against MTB has become a major caveat in developing a novel effective TB vaccine.

### 2.1 Innate Immunity to MTB

**Figure 1:**
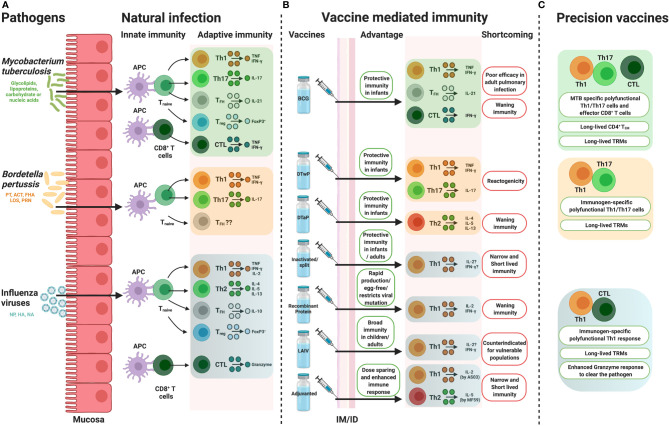
How precision vaccine design takes cues from natural immunity. Overview of the immune responses, portraying from the perspective of conventional T cell biology, induced by **(A)** natural infection, **(B)** pathogen specific vaccines along with their potential advantages and shortcomings and **(C)** possible hypothesis to overcoming the disadvantages by precision vaccine design. APC, antigen presenting cell, Th, T helper, IL, interleukin, TNF, tumor necrosis factor, T_FH_, follicular helper T cell, Treg, regulatory T cells, CTL, cytotoxic CD8^+^ T cell, BCG, Bacille Calmette-Guérin, DTwP, Diphtheria and Tetanus Toxoids along with Whole Cell Pertussis, DTaP, Diphtheria and Tetanus Toxoids along with Acellular Pertussis, LAIV, live attenuated influenza virus, IM, intramuscular, ID, intradermal, IFN, interferon, PT, pertussis toxin, ACT, Adenylate Cyclase Toxin, FHA, filamentous hemagglutinin, LOS, lipo-oligosaccharide, PRN, pertactin, HA, haemagglutinin, NA, neuraminidase, NP, nucleoprotein, TEM, effector memory T cell, TRM, tissue resident memory T cells. Figure is created with BioRender.com.

Emergence of innate immunity is essential between MTB and host which eventually initiate the long term memory responses. Lung resident macrophages, neutrophils, dendritic cells (DCs), and natural killer (NK) cells are the major participants of pulmonary innate immunity. As an intracellular pathogen, MTB first comes in contact with airway epithelial cells and is phagocytosed by lung-resident macrophages (23). During the innate response, phagocytosed MTB can be cleared from the phagosome or induce Th1 type adaptive immunity (**Figure 1A**) *via* antigen processing and presentation (24). After infection, pattern recognition receptors (PRRs) of macrophages recognize pathogen-associated molecular patterns (PAMPs) of MTB. PRRs including Toll-like receptors (TLRs), Nod-like receptors (NLRs) and C-type lectin receptors (CLRs) coordinate multiple signaling cascades (25). TLRs recognize mycobacterial glycolipids, lipoproteins, carbohydrates or nucleic acids as a PAMP. Signaling between MTB’s PAMPs and TLRs activates MyD88 and elicits proinflammatory cytokines or type I IFNs (25). All TLRs use MyD88 for their downstream signaling except TLR3 (26). Involvement of TLR2, TLR4, TLR8 and TLR9 are well documented during MTB infection (27, 28). TLR2, TLR4, TLR7, TLR8 and TLR9 polymorphisms in human are associated with greater exposure to pulmonary MTB infection (25, 28).

After recognition, MTB is eliminated by several pathways including phagocytosis, inflammasome activation, autophagy and apoptosis (29). After infection, macrophages differentiate into either classically activated macrophage inflammatory type 1 ‘M1’ or alternatively activated ‘M2’ macrophages (25). ‘M1’ macrophages are pro-inflammatory and anti-microbial in nature while ‘M2’ macrophages are anti-inflammatory in nature (30, 31) and poor antigen presenting cells (25). Recent research highlights the M1/M2 model after MTB infection (30) though the exact mechanism remains a dilemma (31). MTB DNA is sensed by cGAS-STING pathway and triggers downstream cytokine production and autophagy (32, 33). STING pathway is found to be important for DC activation but unessential for protective immunity (34). DC-SIGN (CD209) acts as an entry point of MTB to DCs (35). CD14^-^HLA-DR^+^DC-SIGN^+^ cells are probably responsible for endocytosis of MTB in humans (36). Major histocompatibility complex (MHC) unrestricted innate lymphoid cells (ILCs), which are enriched in intestinal and alveolar mucosal surface, also play a crucial role in human MTB infection (37). In active TB patients circulatory ILCs in blood were found to be depleted and restored upon treatment. In general, ILCs can module both innate and adaptive immune responses by producing interferon (IFN)γ (by type 1 ILCs), interleukin (IL)-4, IL-5, IL-13 (by type 2 ILCs), IL-17 and IL-22 (by type 3 ILCs) (37). There is evidence that CD117^+^ ILC3 specifically contribute in the immune response to MTB (37, 38). Neutrophils, granulocytes and mast cells have protective roles against MTB (35). Little is known about the exact immune mechanism of neutrophils and mast cells in human MTB infection (25, 35) which might open avenues for further research.

### 2.2 Adaptive Immunity Achieved by Natural MTB Infection

For immunologists to develop a new and more effective vaccine against TB, relevant immunological outputs must be considered to select the optimum formulation, delivery mechanisms and adjuvantation. Prioritization of readouts (e.g., antibody titer, antigen-specific Th1 polarization, T cell polyfunctional activity etc.) will need to either be instructed by correlating and implicating functions following novel vaccine development or by further understanding of the most important immunological outputs following natural MTB infection. T cells have a central role in protective and adaptive immunity and they are the prime candidates to target and trigger effective adaptive immunity. Predominantly, activated antigen presenting cells (APCs) induce proliferation of antigen specific naive CD4^+^ T cells (**Figure 1A**). The mechanism of antigen delivery in humans after MTB infection remains somewhat obscure. Peripheral blood CD1c^+^ cells have been reported to initiate T helper 1 (Th1) polarization in active MTB patients (39, 40). Impairing IFNγ production by CD11c^+^CD1c^+^ conventional DCs in human has been recently characterized (41). In the last 10 years, T cell expansion/contraction kinetics and development of memory signature by T cells have been well established by the studies of CD8^+^ T cell responses against acute viral infection (42–44). These cannot, however, be directly translated to bacterial infection and MTB disease. Working with slow growing pathogens like MTB and lack of tetramers to target antigen specific T cells (42, 45) along with unavailability of transgenic T cell receptor mice are the intricate barriers for T cell readouts. High sensitivity multi-color flow cytometric assay and cytometry by time-of-flight (CyTOF) might shed light on the unexplored phenotype of APCs during MTB infection and are identified here to be essential areas of future research.

Comparative studies with MTB infected individuals demonstrated accumulation of MTB specific TNF^+^ CD4^+^ (46) and polyfunctional (IFNγ, IL-2, and TNF producing) T cells (47, 48). Th1 polarization and IFNγ or TNF production are essential to clear MTB infection (29, 49). IFNγ heightened antimycobacterial defense in macrophages, whereas TNF synergizes with IFNγ to combat against MTB infection (30). A balanced immune response is required, as IFNγ production by T cells has also been implicated to be associated with TB in a potentially age-dependent manner (50). It remains to be determined whether this was a causative or associated observation. However, other CD4^+^ T cell subsets, especially IL-17 (Th17) and FoxP3^+^ regulatory CD4^+^ T cells also contribute to the host resistance against MTB infection (33, 51, 52) (**Figure 1A**). MHC-I-restricted CD8^+^ T cells contribute to defense against MTB infection (47, 53). Synergy between CD8^+^ and CD4^+^ T cells restricts intracellular MTB growth (54). NK cells (55, 56) and B cell responses to MTB infection have also been characterized in the clinical spectrum (38). B cell responses to tuberculosis have had conflicting and potentially negative results in response to infection/disease. Animal models have identified either no effect or delayed pathogenesis from MTB following B cell depletion (57), but animal models can have differential responses compared to human pathology (58), limiting the cross-species conclusions that can be drawn. B cells and antibodies have been functionally implicated in anti-TB immunity. B cells have been observed to accumulate around MTB granulomas in the lungs, induce various cytokines like IL-10 (57), form germinal center-like structures in non-secondary lymphoid tissues that support cell proliferation and restrict TB dissemination and instruct effective T cell responses (57, 59). Further B cell importance in containing TB disease can be observed in the importance of humoral immunity. Individuals repeatedly exposed but uninfected, termed “resisters” (60), have had a significant induction of TB-specific IgG levels and antibodies specific to ESAT-6 and CFP-10 in latent TB can separate from subjects with active TB (59). TB-specific IgA secretion into the lungs has been implicated following infection which could contribute to antibody-dependent cellular phagocytosis and cytotoxicity, activation of complement, direct TB neutralization, stimulation of cell mediated immunity and intracellular identification through tripartite motif containing protein 21 (TRIM21) [as reviewed in (59)]. Donor Unrestricted T cells (DURT) or unconventional T cells, i.e. mucosal-associated invariant T (MAIT) cells, CD1-restricted T cells and γδ T cells play a crucial part in early stage of defense (61, 62) by recognizing non-peptide mycobacterial antigens (33). Tissue resident memory T cells (TRM) proximally located in non-lymphoid lung tissue can protect from TB in various models, independently from Th1 polarization, representing another immune mechanism of interest for protection (63, 64). TRM could also contribute to greater inflammatory damage and additional research is required to understand the involved mechanisms (63). Activated and exhausted TRMs were found to be predominant at infection sites in TB patients (65). A very recent study identified the protective role of IL-17 producing TRM cell clusters in MTB infected human lungs (66). Further *in vivo* observations and correlates of protection to identify which immunological response(s) are essential are needed before development of new vaccines against MTB.

### 2.3 Protective Immunity After BCG Immunization

At present, BCG is the only WHO recommended tuberculosis vaccine indicated for human use in endemic areas. BCG offers infants protective immunity against disseminated tuberculosis (67). Unfortunately, BCG is less efficacious against adult pulmonary MTB infection (68, 69). Some possible hypotheses for the poor efficacy in older persons could be due to the improper induction of memory precursor effectors cells (MPECs) which are capable of generating long-lived memory cells (53, 69). Reinfection could also be a hindrance to BCG efficacy (69, 70). Age-dependent innate immunological responses to BCG have also been identified in a neonatal and adult mouse model (71). Lastly and importantly, BCG strain variants and the degree of viability between MTB strains is now recognized to drive distinct immunological outcomes after BCG vaccination (72, 73), highlighting the importance of the immune system’s ability to recognize viability to elicit strong innate immune responses against vita-PAMPs (74) and should be induced through adjuvantation. This is particularly evident in the as of yet most efficacious new development of a subunit TB vaccine, M72/AS01_E_ with AS01_E_ adjuvantation (75) to induce TLR4 signaling (76) that is otherwise triggered by the vita-PAMPs (77). The 54% efficacy of M72/AS01_E_ was significant for an inactive nonreplicating vaccine (75), particularly compared to the range of 0-80% efficacy for BCG (78), but still highlights an unmet requirement for further vaccine development. Pursuing the development of a new vaccine will not necessarily replace the need for BCG vaccination. Human trials have displayed potential efficacy of a heterologous prime boost with neonatal BCG vaccination followed by adolescent immunization with an alternative protein subunit, H4:IC31, that reduced signatures of long-term infection (79). Understanding the variable responses to vaccination already present in humans is crucial to identify how to design novel and superior vaccines.

As a “self-adjuvanted” vaccine, BCG triggers innate immunity through TLR2, TLR4, TLR8, C-type lectins and Mincle (73, 80) after intradermal vaccination. When co-delivered, BCG can therefore act to enhance the immunogenicity of non-related protein vaccines (81). After internalization by APCs, BCG induces DC maturation (82) and migration followed by antigen 85 mediated production of TNF, IL-1β and IL-6 which promote immune cell activation (80, 83). BCG develops adaptive immunity when antigens are processed by MHC class II and I pathways and activated both CD4^+^ and CD8^+^ T-cell subsets with elevated production of IFNγ (73, 83) (**Figure 1**). BCG stimulates the generation of effector memory T (T_EM_) cells which has been phenotyped as CD44^high^ CD62^low^ CCR7^low^ (84, 85). T_EM_ cells usually engaged in various cytokine production and affected tissue specific homing (86). Induction of humoral responses has been documented 4-8 weeks post vaccination period (80, 87). B cell response triggers the induction of IgG (88) and long-lived memory B cells (89). Induction of TRMs following BCG immunization depends on the route of immunization (90). MTB specific TRM responses are shown to be induced by mucosal BCG immunization (90, 91) rather than intradermal (91) or subcutaneous BCG administration (90). MTB specific TRMs were proven to participate in a protective role upon pulmonary reinfection (92). TLR8 on human monocyte senses live BCG and promotes T follicular helper (T_FH_) cell differentiation (74) and might be a key player in BCG specific humoral responses. Identification of the PRR-mediated immune activation profiles by different strains of BCG could reveal important correlations to various levels of protection and identification of beneficial targets (i.e., antigens, TLRs) for future vaccine development efforts.

### 2.4 Lessons From Natural MTB Infection and Future Vaccine Strategies

Deep understanding of the BCG mediated immune protection, MTB pathogenesis and human immunogenicity are still unclear. TB infection can be categorized into different stages with transmission, development of active disease and continuation to latent TB infection (LTBI) (93). Interestingly, an estimated 9/10 individuals who develop TB-specific T cell responses fail to develop active disease (93), suggesting that there could be immunological mechanisms triggered by TB that may be sufficient to protect from active infection in some individuals (53). This is not sufficient protection, however, as the remaining 1/10 TB-exposed individuals progress to active disease and afterwards, in survivors, progress to LTBI with a lifelong danger of TB reactivation (53, 93). The disease stages are often presented as either active or latent but can be further observed as a disease spectrum (93). Furthermore, a group of exposed individuals, termed “resistors”, have remained uninfected (by tuberculin skin test or IFNγ release assay (IGRA) negative) despite high TB exposure, suggesting some individual’s ability to trigger effective natural immunity (53, 60). Resistors were characterized by IgM, class-switched IgG antibody responses, high accumulation of IL-2 (94, 95) and IFNγ independent T cell responses to the TB specific antigens (60), identifying important mechanisms to induce post-vaccination. This may not however be the entire immune profile required to contain infection, as TB reactivation in HIV individuals has been observed prior to a depletion of CD4^+^ T cells (93). Further studies investigating human TB infection are needed to evaluate which combination of immune mechanisms allow resistors to avoid active infection to identify the essential pathways needed to be induced in a wider population. Meanwhile, adjuvanted multi-antigen TB vaccine development can target induction of multi-antigen and multi-faceted immune responses, triggering IgM, IgG, lung resident TRMs and IFNγ independent immune responses with the goal of overcoming TB’s active impairment of host immunity that otherwise restricts immunity and prevents sterilizing immunity.

A single highly immunogenic antigen that can induce sterilizing protective immunity against all disease stages of TB in a varied population has not yet been discovered and may not be possible. Selection of which vaccine antigen to induce protection from can be guided by observing natural immune responses to MTB, through focusing on pathways that can convey partial immunity. MTB encodes a wide variety of proteins with 4,019 open reading frames, one fourth of which are hypothetically expressed proteins that need to be experimentally verified (96, 97). MTB has evolved to actively produce factors inhibiting innate immune responses (98), identifying previous selective pressures that protected from infection. Identifying natural protection from disease can highlight effective vaccination targets. This highlights the need to target multiple antigens, potentially from multiple disease stages, so that effective immunity is induced prior to infection that spans the breadth of TB disease. The approach of selecting immunodominant antigen targets to vaccinate against is not always effective though (99) and targeted induction of protective immune responses may be required to maximize protection.

Numerous efforts have been attempted to replace the century-old BCG vaccine. Various candidates have made it to clinical trials, with some efficacy observed in completed trials and others ongoing as of 2021. These vaccines fall into various vaccination categories, including adjuvanted antigen (M72:AS01_E_, H56:IC31, ID93:GLA-SE and GamTBvac), viral vectors with TB antigens (TB/FLU-04L, MVA85A and Ad5Ag85A), inactivated vaccines (*M. vaccae*, *M. indicus pranii*, DAR-901 and RUTI) and live-attenuated (VPM1002, BCG revaccination and MTBVAC) (53, 100, 101). M72:AS01_E_ particularly had similar efficacy to BCG’s 50% reduction in IGRA conversion, with a 50% reduction in disease (75). This level of protection was however insufficient at overcoming BCG’s protective effect and indicates an unmet need for TB prevention. Ongoing vaccine development includes evaluation of various adjuvants and alternative antigen and adjuvant delivery technologies (102). The recent development of mRNA encapsulated in lipid nanoparticles to protect from SARS-CoV-2 has garnered an interest in evaluating the technologies’ potential in TB prevention (103). Modern vaccinology solutions are being evaluated to garner greater protection from TB than the century-old BCG, but significant efforts are still needed.

## 3 *Bordetella pertussis*

Pertussis or whooping cough caused by highly contagious *Bordetella pertussis* (BP) is a major health concern in the infant population. Pertussis was the largest cause of infant morbidity and mortality during the first few decades of the 20^th^ century (104). Despite the routine immunization with whole cell pertussis (wP) and acellular pertussis (aP) vaccine, pertussis remains resurgent. Lower efficacy of aP comparative to reactogenic wP and waning of protective immunity after aP immunization might be an effect of increasing pertussis incidence in developed countries. Reviewing and highlighting natural immunity as well as differences in immunological stimulation by aP and wP could identify the significant pathways needed for long term protection.

### 3.1 Innate/Adaptive Immunity to Pertussis

BP uses wide variety of virulence factors as a PAMPs involving Pertussis Toxin (PT), Adenylate Cyclase Toxin (ACT), Filamentous hemagglutinin (FHA), Pertactin (PRN) and Lipo-oligosaccharide (LOS). FHA helps BP in adherence to respiratory mucosa and ACT helps in invasion while PT detours the phagocytosis of BP by APCs (105). After colonization to respiratory epithelium, BP initiates the arm of innate immune responses which later helps to shape the adaptive arm. Different classes of PRRs of macrophages including TLRs, NLRs and CLRs recognize PAMPs of BP (106), therefore, with biomimicry, adjuvantation inducing similar pathways may improve efficacy. Clinical isolates of BP generally enhance TLR2 and TLR4 signaling (107). PT and LOS activate TLR2 and 4 signaling in human (106, 108), trigger IL-12 production (109) while FHA acts as a TLR2 ligand (110) and interacts with ACT (111) to play a crucial role in natural immunity. Destruction of phagocytosed BP by macrophage activated IFNγ and IL-17 is debatable (105). The specific role of PRN in human BP pathogenesis remains unclear though it helps to overcome neutrophil-mediated clearance in mice (112). Targeting any, or multiple, of these BP-produced proteins could be valid immunological targets.

After BP infection, professional APCs prime the differentiation and proliferation of naïve T cells into effector and memory T cells. While the roles of CD4^+^ T cells in BP infection are well documented (**Figure 1**), the specific role of CD8^+^ T cells is less clear (113). Generally, naïve T cells differentiate into TNF and IFNγ producing Th1 (114, 115), and IL-17 producing Th17 cells after natural BP infection (116). Th1 and Th17 cell mediated immune responses in bacterial clearance and protection are demonstrated in mice and baboon infection models (116). Animal study revealed that tissue resident memory (TRM) T cells (phenotypically defined as CD4^+^CD69^+^) confer long term protection against BP (117, 118). The role of TRM in human BP infection remains unexplored (119). Macrophage dependent IFNγ producing CD56^+^ NK cells activation in human is reported very recently (120). IL-17 dependent neutrophil recruitment (121) and existence of Foxp3+ Treg cells in lungs of BP infected mice (108) were documented. BP specific IgG and IgA might have a role in clearance of bacterial load by neutralizing BP toxin or by opsonization (122). Further studies are necessary to dissect the protective role of such cells in human BP infection, so that important correlates of protection can be identified post-vaccination.

### 3.2 Whole Cell and Acellular Vaccine-Induced Immunity

Emergence of BP infection in the US population was dramatically reduced (99%) by the introduction of alum containing wP vaccine in 1950s (123, 124). In the USA during mid 1990s, alum-formulated aP replaced wP based formulations. The main driving factor was the desire to further reduce the rare incidents of febrile seizures in infants post vaccination, while nearly always recoverable, were associated with wP induced local and systemic reactogenicity (124, 125). The formulation of alum adjuvanted Diphtheria and Tetanus Toxoids along with Acellular Pertussis (DTaP) vaccine is variable, ranging from a mixture of virulence factors like PT alone or with FHA, PRN and/or fimbriae serotypes (FIM 2/3) (112, 126, 127). Despite successful DTaP vaccination, resurgence of pertussis was observed in developed countries in the first decade of 21^st^ century (128). Lower efficacy and waning immunity in response to DTaP has become a major concern (129).

Formulation of alum adjuvanted Diphtheria and Tetanus Toxoids along with Whole Cell Pertussis (DTwP) vaccine acts differently and induces distinct immunity profile in humans than DTaP. DTwP which is derived from killed BP, is enriched with several prominent antigens (130) along with exogenous TLRs agonists. wP primed BP specific CD4^+^ T cell immunity is Th1/Th17 biased which mimics cellular immunity profile after natural infection (128, 131) (**Figure 1**). The protective role of BP specific IFNγ producing CD4^+^ T cells (Th1 cells) and IL-17 producing CD4^+^ T cells (Th17 cells) have been well demonstrated in animal models (119). In mice, BP infection usually induces IgG2a/2b antibodies which triggers cell mediated and IgG induced humoral responses (132). All IgG subclasses (IgG1/2a/2b and 3) were induced by wP immunization, which clearly explains why wP immunization is linked to Th1/Th17 polarization (132). aP evokes IgG1 induction (132) and induces IL-4, IL-5 and IL-13 producing CD4^+^ T cells (Th2 cells) (**Figure 1B**) which does not confer protective immunity against nasal infection and transmission (119). wP induced respiratory TRM cells also participate in protective immunity in mice which were found to be absent followed by aP vaccination (118), but some evidence indicates that there may be a specialized role for Th17-polarzied TRM cell subsets in the control of nasal infection in aP immunized mice (133). Benchmarking wP versus aP immunization in human is challenging as in developed countries only aP vaccine is licensed. Interestingly, a recent study between wP and aP primed individuals showed that only wP prime evoked BP specific CD4^+^ T cells after aP boost (134). aP prime followed by aP boosted donors exhibited increased Th2 related cytokines, reduced IFNγ and IL-17 production, defective T cell memory expansion and lower T cell proliferative capacity (134). Data from multiple cohort studies also proven that DTaP induces Th2 skewed immunity whereas DTwP primed Th1 biased immunity in human (119, 135, 136). Moreover, aP vaccines do not inhibit colonization and transmission of the disease (137). Thereby, aP vaccines do not confer herd immunity in population (138). These outcomes partially elucidate the resurrection of pertussis in developed countries though further study is necessary to dissect the host immune response after BP pathogenesis and vaccination.

### 3.3 Overcoming Waning Immunity and Lessons From Natural BP Infection

Overall, the two main limitations of the current aP vaccine are its relatively short duration of effectiveness and the lack of local immunity in upper respiratory tract. Importantly, a study of infant olive baboon (*Papio anubis*) challenge model has revealed that convalescent animals are fully protective from subsequent bacterial challenge regarding upper-airway colonization, whereas aP vaccinated animals showed bacterial shedding (137). Thus, it is a reasonable approach to find cues to overcome these limitations in studies from natural infections and from more immunogenic wP vaccine. As summarized above, aP vaccine-induced immune responses can be distinguished from wP and natural infection-induced immune responses with its 1) weak Th1/Th17 polarizing CD4^+^ T cell immunity, 2) low induction of tissue resident memory T cells and possibly 3) low functional antibody responses. Therefore, to hypothetically overcome the waning effectiveness of acellular vaccine, development and evaluation of a next generation DTaP vaccine is required which should prime Th1/Th17 polarization. Adjuvantation using TLR agonists or STING (stimulator of interferon genes) ligands might be a smart choice (13, 139, 140). Alum adsorbed TLR7 agonist adjuvanted aP formulation primed BP specific Th1/Th17 polarization and humoral responses in adult mice than traditional aP formulation (141). Protection against BP aerosol exposure was achieved in TLR7 agonist adjuvanted aP formulation immunized mice group, similar to that triggered by wP (141). Preliminary study with a novel TLR7/8 agonist CRX-727 (UM-3003) formulated DTaP (142), a collaborative work between University of Montana/Inimmune and Precision Vaccines Program/Boston Children’s Hospital, revealed enhancement in early life immunogenicity and overcomes neonatal hyporesponsiveness to DTaP (143). Overcoming neonatal hyporesponsiveness is a major caveat in development of next generation acellular pertussis vaccine. Neonatal cells are Th2 biased due to hypomethylation in Th2 locus (122). Therefore, restoration of Th1 predominance could be achieved either by TLR7/8 adjuvantation or by live vaccine strain like BCG. TLR2 ligand from BP itself, is capable of driving innate immune responses along with protective homologous immunity upon respiratory challenge (144). Long lasting protection against nasal exposure in mice was reported by a novel adjuvant combination including STING and TLR-2 (145). aP formulated with STING and TLR-2 skewed Th1/Th17 polarization and triggers IL-17^+^ TRMs (145). TLR9 agonist adjuvanted DTaP confers enhanced protection than DTaP alone or TLR4 adjuvanted formulation in mice (146).

A promising live attenuated BP vaccine strain (BPZE1) is in human phase 2 trial (127, 147), and proven beneficial in mice and infant baboon models (148). Early protection in mice induced by BPZE1 is independent of the adaptive immune system but depends on TLR4 signaling pathway (149). GamLPV, another live intranasal BP vaccine strain, is also in phase 1/2 trials (112). Using live BP strain as a vaccine, has certain advantages- i) a live strain has a variety of BP antigens compared to aP vaccines which only contain up to 5 antigens, ii) live attenuated strain mimics natural infection and provides sterilizing immunity. Improved vaccine efficacy should be achieved by live BP vaccine strain as TLR8 on innate immune cells senses live bacteria (74) and triggers T_FH_ differentiation which is beneficial for inducing strong humoral immune responses (74). Lately, a first controlled human study with BP infection was conducted by a European collaboration to get the insight of microbiological and immunological features of BP pathogenesis (150, 151). This novel approach will allow us to dissect the mechanism of protection in the context of natural BP infection (151).

## 4 Influenza

Influenza viruses belong to the *Orthomyxoviridae* family which represents enveloped viruses, with genome consisting of segmented negative-sense single-strand RNA segments (ssRNA). These ssRNA are tightly surrounded by nucleoprotein (NP), which along with glycoproteins such Haemagglutinin (HA) and neuraminidase (NA) are the viral components detected by antibodies, and also define the subtype of the virus (152). Although, there are 4 genera of this family namely A, B, C and D; only influenza A and B are clinically relevant in humans and responsible for causing seasonal epidemics (known as the flu season) almost every winter across the world (153). Influenza infection in humans is initiated in the respiratory tract and in most cases is limited to this organ. Oral or nasal entry of the virus is initially countered by the mucus lining the respiratory epithelium. Upon successfully breaching the mucosal layer, the virus can attach and invade the respiratory epithelium where it can spread to both non-immune and immune cells [such as macrophages and dendritic cells (DCs)] in the respiratory tract (152, 154).

### 4.1 Innate Immunity to Influenza

Innate immune system forms a formidable barrier as part of the defense mechanism against influenza virus (152). Detection of the virus through the innate immune system occurs *via* at least three distinct classes of the PRRs; TLRs (including TLR3, TLR7 and TLR8), retinoic acid-inducible gene I (RIG-I) and the NOD-like receptor family member NOD-, LRR- and pyrin domain-containing 3 (NLRP3) (155). TLR3 detects virus-infected cells, and TLR7 (and TLR8 in humans) detect viral RNA endocytosed by the sentinel cells (i.e., cell-extrinsic recognition). However, RIG-I and NLRP3 are responsible for detection of virus present within the cytosol of infected cells (i.e., cell-intrinsic recognition). Lack of innate sensors and signaling pathways in mice challenged with high doses of influenza A virus has been demonstrated to result in mortality since the host succumbs to infection. However, with sub-lethal doses or inactive virus the host is able to survive the infection by mounting a protective adaptive immune response. Such studies have also expanded our understanding of the viral sensors that link innate recognition to adaptive immunity (152), which is an important progression in being able to model the disease *in vivo* and to test efficacy of vaccination and adjuvantation in a way that may mimic the natural pathways triggered by influenza infection.

Properly modelling influenza infection *in vitro* can be difficult. This is partly due to influenza virus-infected cells not generating dsRNA (156) due to the activity of cellular RNA helicase (157). Therefore, TLR3 likely recognizes unidentified RNA structures present in dying virus-infected cells that have been phagocytosed (158), changing the immunological signaling compared to *in vivo* observations. Activation of TLR3 in human respiratory epithelial cells (which constitutively express TLR3) is known to induce production of pro-inflammatory cytokines upon influenza virus infection but can also lead to pathology (159–161). Further, *Tlr3^-/-^* mice survive longer than their WT counterparts (despite higher viral loads in the lungs). However, viral challenge in *Tlr3^-/-^* mice leads to reduced chemokine expression and infiltration of leukocytes and CD8^+^ T cells in the lungs (160). Surprisingly, with sub-lethal doses of influenza virus infection these mice are able to generate normal antibody responses as well as CD4^+^ T cell and CD8^+^ T cell responses, suggesting TLR3 is dispensable for generating T cell immunity (162). Thus, although TLR3 is crucial to curb viral replication, it simultaneously promotes the recruitment of innate and adaptive responses that result in damage to the host. Although TLR3 signaling can be damaging during host-mediated inflammation to disease, it may be beneficial to induce during vaccination to amplify protective immune responses.

In plasmacytoid dendritic cells (pDCs), independent of viral replication, TLR7 recognizes endocytosed ssRNA genomes within the influenza virion (152). Downstream of TLR7 signaling transcription factors are activated [either nuclear factor-κB (NF-κB) or IFN-regulatory factor 7(IRF7)] which promote expression of pro-inflammatory cytokines and type I IFNs, respectively. The role of TLR7, specifically in inbred mice, in innate defense remains unclear since contradictory studies have shown that TLR7 could be dispensable against high-dose influenza virus challenge. However, studies with sublethal doses of influenza virus have demonstrated pivotal role of TLR7 in eliciting robust antibody response, but not T cell responses. Thus, TLR7 expression is crucial for antiviral responses against influenza virus *via* recruitment of B cells to elicit antibody production. On the contrary, although TLR8 is expressed in human monocytes and macrophages, and its activation results in IL-12 but not IFNα, however the exact role of TLR8 in influenza virus infection remains unclear.

### 4.2 Adaptive Immunity to Influenza

Although innate immunity is essential for restricting viral replication, the adaptive immune responses are critical for eventual viral clearance, recovery and protection from reinfection. This has been established by studies showing that influenza virus challenge in immunodeficient mice leads to significantly higher mortality than healthy controls (163, 164). Importantly, the HA and NA proteins are key targets of the adaptive immunity (165).

#### 4.2.1 Humoral Responses

The mechanisms involving the humoral responses towards influenza virus have been well elucidated in recent decades. During infection, naïve B cells in the mesenteric lymph nodes (mLN) encounter the influenza virus antigen and differentiate into antibody-forming cells (AFCs). Accordingly, B lymphocyte-deficient μMT mice showed high susceptibility to influenza virus infection compared to WT mice (166, 167). The B-cell response against influenza virus commences ~3 days post infection, meanwhile the anti-influenza virus IgG secretion begins by day 7. Systemic AFCs are first detected around 6-7 days post-infection (168), while the maximum number of B cells in bronchoalveolar lavage fluid (BALF) is observed around day 10 post-infection in mice (169). In mice, there is tissue-specific response of AFCs whereby they produce IgG and IgM in the lungs, and IgA in the upper respiratory tract (170).

The HA and NA proteins of the influenza virus are responsible for viral entry and release, therefore antibodies specific to these antigens are crucial for protective immunity. HA-specific antibodies result in viral neutralization through binding to the HA globular head and inhibiting the attachment of the virus to the host cell’s surface (171–173). However, NA-specific antibodies inhibit the enzymatic activity of NA and instead block viral replication (174, 175). Further, M2 proteins have also been shown to be target of specific antibodies and passive transfer of M2-specific antibodies can provide protection against viral replication (176). Interestingly, NP-specific antibodies also facilitate resistance to influenza virus, despite targeting an internal influenza virus protein (177). In addition, the influenza virus-specific antibodies also mediate antibody-dependent cell cytotoxicity and Fc receptor-mediated phagocytosis, and thus significantly contribute to the clearance of infected cells (178). These antigen targets have been classically and effectively targeted with seasonal vaccinations against influenza, however efficacy per season has at times been insufficient (179). If there is no change to the antigen targeted, based on historical strategies, an alternative formulation or adjuvantation would be required for amplified protection.

#### 4.2.2 Cell-Mediated Immune Responses

Naïve CD4^+^ T cells recognize the viral antigens presented by the MHC class II proteins on APCs, and subsequently differentiate into several types of helper T cells depending upon the cytokine milieu. This leads to activation and differentiation of antibody-producing B cells *via* support from the activated CD4^+^ T cells, and induction of CD8^+^ T cells responses (**Figure 1**). In mice, the peak CD4^+^ T cell response is observed at 10 days post influenza infection (180). Adaptive transfer of effector CD4^+^ T cells isolated from mice infected with influenza demonstrated enhanced survival of recipient mice challenged with the virus (180). In particular, the cytokine milieu resulting from influenza virus infection is polarized towards generation of Th1 cells (181), which produce IFNγ, TNF and IL-2. This promotes activation of macrophages, production of IgG2a and IgG3 isotypes antibodies from B cells (182), and induction of cellular immune responses. Additionally, Th2 responses are also induced post influenza infection resulting in production of IL-4, IL-5 and IL-13 and isotype switching of B cells to produce other antibody isotypes i.e. IgG1 and IgE (181). However, survival after influenza virus infection is primarily correlated with Th1 compared to Th2 cells (183). Studies using influenza-infected mice have shown involvement of CD4^+^ T populations in perforin/granzyme-mediated cytolytic activity (181, 184, 185). Additionally, influenza virus infection leads to robust Tregs that mediate immunosuppression and tissue repair *via* amphiregulin and IL-10 (186, 187), and depletion of Tregs results in a reduction of the influenza-specific T_FH_ response (188). Thus, highlighting the important role of Tregs in host protection during and after influenza virus infection.

CD8^+^ T cells recognize influenza viral antigens presented by MHC class I proteins on the surface of APCs and are crucial for viral clearance and host protection. CD8^+^ responses peak around 8^th^ day post-infection in the mLN and at 10^th^ day in BALF (169, 189). Mice lacking CD8^+^ T cells (β2-microglobulin-deficient mice) upon influenza viral challenge demonstrate delayed viral clearance and severe mortality (190). Activated cytotoxic CD8^+^ T cells (CTLs) eliminate virus-infected cells *via* cytolysis by producing perforin to permeabilize their membrane and secreting granzyme to induce apoptosis (175). In addition, CTLs can also kill infected host cells *via* TNF receptor family-dependent pathways. CTLs express the Fas ligand (FasL) which binds to Fas on target cells and this interaction induces apoptosis *via* caspase-cascade activation. CD8^+^ T cells also express TNF-related apoptosis-inducing ligand (TRAIL) responsible for CD8^+^ T cell-mediated cytotoxicity (191). Effector CD8^+^ T cells produce TNF and IFNγ in the lungs, which aids the viral defense mechanisms (192–194). Remarkably, these cells are also major producers of IL-10 which regulates pulmonary inflammation during response to the influenza virus infection. Moreover, even with sublethal influenza virus challenge blockade of IL-10 signaling augments pulmonary inflammation and lethal injury (195).

### 4.3 Currently Licensed Influenza Vaccines

#### 4.3.1 Whole Inactivated Virus Vaccines

Although currently not in use due to high reactogenicity, whole inactivated virus vaccines are the easiest to generate and thus have been extensively used in humans as well as studied in animal models. Depending upon the inactivation method employed, inactivated viruses can represent antigens of live virus relatively well. Thus, these vaccines may potentially retain activity of HA and fusion of HA as well as NA activity. Additionally, whole inactivated virus vaccines also contain viral RNA which can activate multiple PRRs including TLR3, TLR7, TLR8 and RIG-I (196), thus resulting in a self-adjuvanting effect. These vaccines can induce a relatively balanced immune response, leading to a response to both HA and NA in humans as well as animal models, and relatively high seroprotection rates (>85% in humans) (197–200). However, in terms of seroprotection and geometric mean HA inhibition (HAI) titers, whole inactivated virus vaccines did not demonstrate any additional benefit compared to split virus or subunit vaccines (201) and understanding of cell mediated responses in humans remain unclear.

#### 4.3.2 Split Virus and Subunit Vaccines

Split virus or subunit vaccines are manufactured from whole inactivated virus which are treated with detergent and further purified. These vaccines are classified based upon the viral components incorporated after downstream purification process, preparation containing parts of the viral membrane carrying HA and NA, referred to as split virus vaccine, and with almost pure glycoprotein referred to as subunit vaccine. Notably, most of the viral RNA is removed during the purification process, which leads to reduced reactogenicity but might also leads to weakened immunogenicity. Moreover, the structural integrity of HA and NA proteins, as well preservation of crucial antibody-binding epitopes in these vaccines remains unclear. The immune response after vaccination with a split virus or subunit vaccines is typically targeted towards HA since the NA content of these vaccines is neither standardized nor detectable in certain cases (200).

The breadth of the humoral response to whole inactivated virus vaccines, split virus and subunit vaccines has been widely evaluated. In addition, these can induce cross-reactive antibody responses to historic virus strains in adults with pre-existing immunity (201, 202). However, they fail to induce significant titers of cross-reactive stalk-specific antibodies. Importantly, current split and subunit vaccines are inefficient in inducing cross-reactive CD8^+^ T cells, which would otherwise be elicited by natural influenza virus infection (203). In addition, vaccine effectiveness is markedly reduced due to antigenic mismatches between circulating virus and vaccine virus strains. Therefore, they tend to induce responses which are remarkably narrow and strain specific.

#### 4.3.3 Recombinant Hemagglutinin Vaccines

Recombinant HA vaccines contain only HA, therefore the immune response specifically targets HA. These vaccines typically contain higher doses of HA (up to 45 μg per strain) compared to split or subunit vaccines, however the antibody responses generated are at least comparable to that induced by whole inactivated, subunit or split virus vaccines (204). Although recombinant HA vaccines were licensed only recently, they have been extensively studied in human clinical trials. Moreover, growing literature suggests that recombinant HA vaccines work particularly well in the elderly by inducing broader responses and providing better protection (205, 206). In a recent study, recombinant HA vaccine demonstrated superior HA-specific both antibody and CD4^+^ T cell responses in adult human cohorts when compared with inactivated influenza vaccine (207). Notably, since the HA is expressed recombinantly, these vaccines overcome mismatches that occur in regular vaccine seed strains due to adaptation of the influenza virus while cultured in eggs (208).

#### 4.3.4 Live-Attenuated Virus Vaccines

The vaccines discussed above are typically either administrated intramuscularly or intradermally, however it remains unclear whether these routes of administration optimally induce mucosal immune response in the upper respiratory tract (209). The mucosal immunity induced through these vaccination routes in humans could be the result of priming by natural influenza virus infection. In contrast, live-attenuated virus vaccines which use a weakened (or attenuated) form of the virus are administered intranasally and promote replication of live attenuated influenza virus (LAIV) in the upper respiratory tract. Thus, the immune response to LAIVs is multifaceted which does not necessarily involve a serum antibody response, and LAIVs have been licensed based upon efficacy trials that measure protection rather than correlates of protection. Additionally, a study comparing long-term systemic and secretory antibody responses in children who were administered live, attenuated or inactivated influenza vaccine demonstrated LAIVs induce antibody responses which persist significantly longer (210). Multiple studies have shown enhanced efficacy of LAIVs in young children compared to inactivated vaccines (211, 212) whereby LAIVs have been shown to induce diverse T-cell responses by inducing CD4^+^, CD8^+^ and γδ T cells (213). In addition, in adults with extensive and partially cross-reactive pre-existing influenza immunity LAIV has been shown to boost secretory IgA responses to HA and non-HA antigenic targets expressed by circulating influenza strains (214).

#### 4.3.5 Adjuvanted Influenza Vaccines

Despite current influenza vaccines being immunogenic, evolution of the virus can reduce efficacy of the vaccines. Adjuvanted influenza vaccines incorporate adjuvants which can boost immune response. This is particularly beneficial for influenza vaccines administered during a pandemic when a rapid response is required or for use in vulnerable populations, such as infants and the elderly. Up until the early 21^st^ century, alum remained the only adjuvant included in licensed vaccine formulations, until, in 2015 MF59 (squalene emulsion) was incorporated into a licensed influenza vaccine designed for enhancing efficacy for the elderly populations in the USA (215). MF59 triggers effective and safe immunogenicity *via* Th2 skewed immune responses (216) and induces chemokines and cytokines, such as CCL2, CCL3 IL-8, and IL-5 (217), which enhance vaccine efficacy by triggering memory B cells and vaccine strain specific CD4^+^ T cells (218). Adjuvant system (AS) 03 is an oil-in-water adjuvant which acts similar to MF59 (215). MF59 and AS03 adjuvants also showed heterologous immune responses against non-vaccine influenza strains in human subjects (215). Thus, vaccine adjuvants embrace a numerous potential to achieve a universal flu vaccine by inducing cross-protective immunity.

### 4.4 Lessons From Influenza Infection and Future Vaccine Strategies

Development of a “universal vaccine” for influenza is challenging because seasonal vaccines lack efficacy against most circulating influenza strains due to antigenic variability. One of the current approaches for influenza vaccine development is to mimic the natural exposure *via* attenuated viral infection or viral vector administration which triggers natural immunity. Live attenuated licensed vaccine FluMist showed 70-90% efficacy over placebo in 1^st^ year and 47-80% efficacy in 2^nd^ year after immunization (219). FluMist promotes virus specific lung TRM and establishes long-term protection compared to inactivated attenuated formulation (220) and was proven safe in a surveillance study (221, 222). CD8^+^ lung TRMs confer frontline defense and accumulate after viral reinfection, but response may wane overtime (223). As such, in depth understating of targeting and optimizing the effector activity of TRMs offers significant opportunity to achieve long-lasting protection (224). Attenuated influenza virus with adjuvant [MER4101, (cH8/1N1, H5/1N1)] or non-adjuvant (GHB11L1, GHB16L2, M2SR) and viral vector (Ad4-H5-Vtn, MVA-NP+M1, ChAdOx1-NP+M1, VXA-A1.1, GamFluVac) candidates are entered in human clinical trials in the last decade and currently in progress (225) to achieve an improved influenza vaccine. The “unnatural immunity” (16), which does not occur naturally may be achievable by triggering antibodies which do not elicit during the course of natural infection. Such lack of preexisting immunity could be restored by the adjuvantation approaches (226). TLR3 agonist- (dsRNA), TLR4 agonist- (MPLA, GLA-AF, ND002), TLR5 agonist- (Vax128), TLR7 agonist- (Imiquimod) and TLR9 agonist- (CPG 7909) adjuvanted vaccine approaches are already in clinical trial (227). Of note, synthetic TLR7 agonist adjuvant, Imiquimod, induced improved influenza specific homologous and heterologous immunogenicity in a phase 3 trial (228). Micro and nano emulsion formulations like AS25, AS50, SE, Montanide ISA-51 are in phase 2 clinical trial and drove increased influenza specific immune responses (227). Immune protection could be boosted by the production of broadly neutralizing antibodies against influenza viruses by designing DNA vaccines (229). Recently developed mRNA-based vaccines have demonstrated great promise after proven efficacious against severe COVID-19 outcomes, including hospitalization and death. HA based mRNA vaccines are in clinical trials, as reviewed in (230) and may have some advantages over conventional inactivated influenza vaccines (231–233). Another approach is to develop a next generation influenza vaccine which triggers heterosubtypic or heterologous immunity (234). Such broad spectrum or non-strain specific vaccine design is feasible by identifying conserved protein regions shared among pre-pandemic and circulating strains *via* epitope mapping (235, 236). Identification of broadly reactive antigens can be considered as a reverse vaccinology approach which is going to be the most powerful tool for next generation vaccine designing (235). Newborns are particularly at increased risk of severe disease following influenza infection since they may be unable to mount an effective immune response to clear infection. However, a more in-depth understanding of protective responses generated during natural influenza infection can guide development of vaccines which illicit broader responses with limited reactogenicity in these vulnerable populations (237).

## 5 Toolkit for Precision Vaccines to Mimic the Natural Immunity

**Figure 2:**
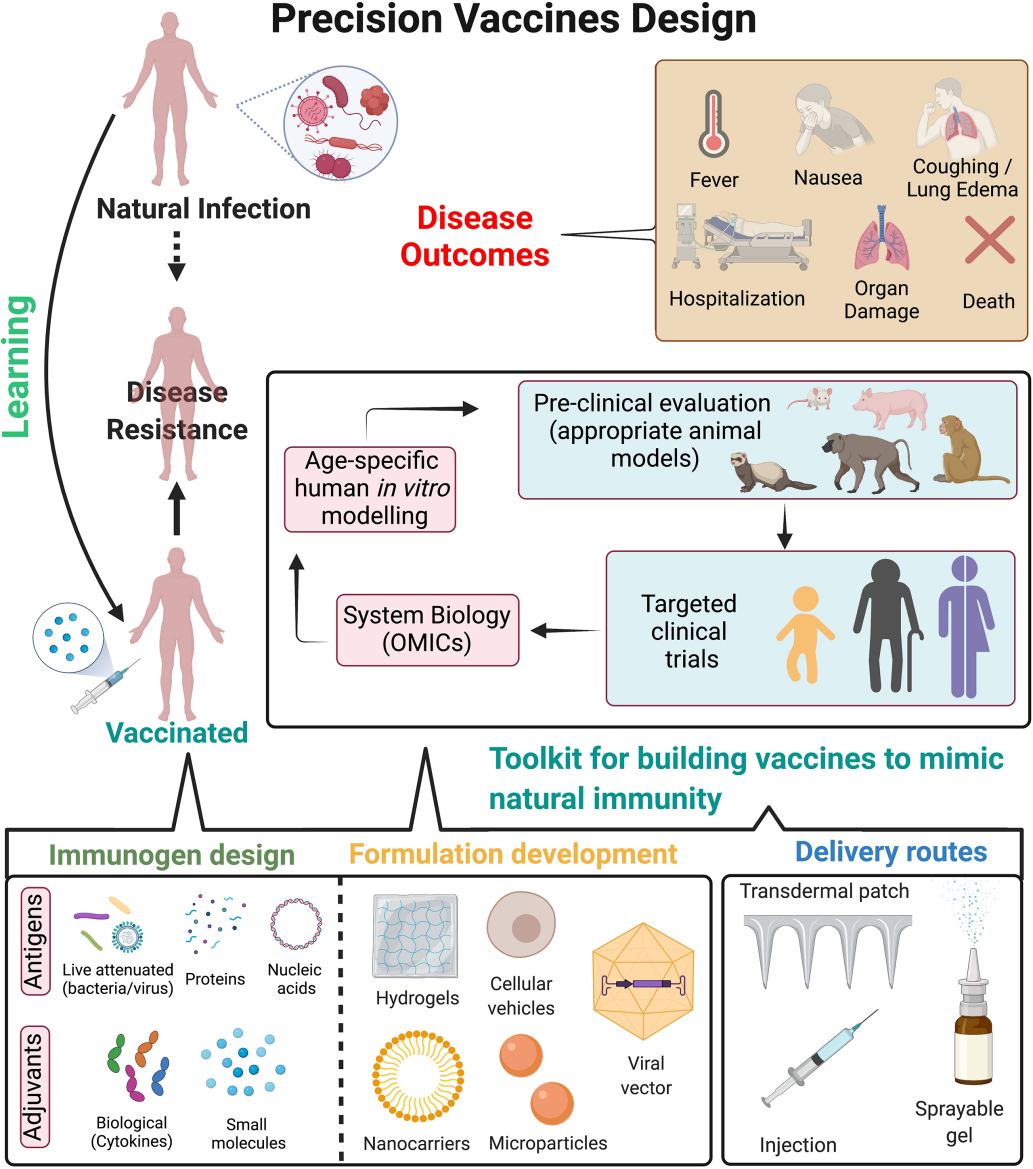
Precision vaccines design. Overview of strategies to design precision vaccines which can effectively mimic immunity against pathogens as observed post natural infection. Natural infection often leads to immunological imprinting which provides long-term immunity and disease resistance to future exposure of pathogens. However, it can also lead to detrimental effects to the host as observed in disease outcomes. On the contrary, vaccination provides disease resistance but may be associated with waning immunity, insufficient protective immune response either in vulnerable populations or to prevent disease transmission, contraindications for immunocompromised hosts etc. Precision vaccines can guide the development of next generation vaccines by incorporating a toolkit for building vaccines to mimic natural immunity, which includes: i) immunogen design (such as antigens, small molecule TLR or other adjuvants, biological adjuvants such as cytokines); ii) optimizing formulations for targeted delivery to antigen presenting cells which can lead to subsequent long-lasting adaptive immune response (approaches such as hydrogels, cellular vehicles, nanocarriers and microparticles etc.); iii) optimizing delivery routes for enhancing immune response and mimicking the natural exposure to the pathogen (such as transdermal patch, injection site, sprayable gels for nasal routes) and iv) pre-clinical evaluation of vaccine formulations in appropriate animal models can be followed by clinical evaluation in distinct populations. System biology approaches from such clinical trials may be helpful to dissect age- and population specific vaccine-induced cellular and molecular signatures which correlate with protective immunity. Further, usage of human *in vitro* modelling may accelerate and/or expand hypothesis testing and selection of population specific adjuvants. These approaches can lead to precision vaccines tailored for long-term disease protection while abating disease outcomes associated with natural infection. Figure is created with BioRender.com.

Based on current understanding of natural infection of MTB, BP and influenza, there are a number of strategies to mimic the immunological manifestation and incorporating it to develop the next generation vaccines (**Figures 1C**, **2**). One major caveat relevant to all vaccination strategies targeting MTB, BP and influenza is waning immunological memory. One approach to bypass it by triggering polyfunctional T cell responses or by inducing long-lived TRMs (**Figure 1C**). Clonal expansion of antigen specific memory T cells mainly depends on the interaction with antigen bearing DCs (238). Unique DC subsets can orchestrate these signals into appropriately regulated adaptive immune responses. Therefore, reprogramming the DC phenotypes (which must mimic the immunophenotype after natural infection) by precision vaccines would be a smart choice. Another strategy would be the fine tuning of the antigen specific T cell proliferation during their developmental phase by using precision vaccine technology (238, 239), such as TLR agonists-based adjuvantation, many of which have the promising ability to drive phenotypic shifts of Th populations (140, 142). Overall, precision vaccinology can offer the fine tuning or reprogramming of DCs or APCs and antigen specific T cells. Antigen and adjuvant discovery, immunoengineering, vaccine delivery and more importantly increased understanding of human immune responses *via* dissecting the components of natural infection are fueling a revolution in vaccinology and guiding the development of precision vaccines (3, 240), especially for vulnerable infants (241) and older adults (242).

### 5.1 Immunogen Design

Conventional vaccine design (live attenuated bacteria/virus-based or protein-based vaccines) provides durable protection against several diseases. However, there remain major obstacles to vaccine development against a variety of pathogens, especially those better able to evade the adaptive immune response. Furthermore, for emerging virus vaccines, the primary hurdle is not the effectiveness of conventional approaches but the urgency for rapid development and large-scale deployment (243). Thus, as observed during the recent COVID-19 pandemic, the development of versatile vaccine platforms has become important (244). Vaccine platforms such as mRNA-based vaccines only require sequence information to trigger vaccine development, thereby increasing the flexibility to adapt vaccines to antigenic changes in circulating as well as new emerging strains. This aids in pre-emptive and reactive vaccine design, moreover faster development and manufacturing options, ultimately enhancing our ability to rapidly respond to emerging viruses (244). Lessons from natural infection can aid these novel vaccine platforms by precisely guiding the selection of immunogen as well as individual specific adjuvant for a more effective and durable vaccine response (**Figure 2**).

### 5.2 Formulation

Recent advances in the field of immunoengineering, which are evolving alongside vaccinology, have begun to greatly influence vaccine formulation design (245, 246). Due to the disparities in mechanisms of action of various non-adjuvanted and adjuvanted vaccines, vaccine formulation development has become a major consideration for vaccinologists and pharmaceutical companies (247). These factors include (1) physicochemical characteristics of the formulation, (2) adjuvant chemical structure and (3) short- and long-term formulation stability (246). Specifically, vaccine delivery systems have now evolved to mimic the shape, size, and surface chemistry of pathogens (248), which are often referred to as “pathogen-like particles”. These vaccine formulations can thus allow control of antigen and adjuvant biodistribution, regulation of vaccine uptake by APCs, optimized triggering of antigen-specific B cells, and precisely influence vaccine kinetics (249).

### 5.3 Delivery Route

Most current vaccination procedures utilize needles and syringes which are administered through the intramuscular route of injection. However, studies in the recent decades have suggested that skin and mucosal membranes which are not accessible by conventional needles, are however the ideal targets for vaccine delivery (250, 251). The shortcomings of injections have led to active research and development for needle-free methods of immunization. Additionally, these alternate methods also reduce the risk of exposure to needle-borne infections, lower toxicity and economic costs, and ultimately enhance safety and reproducibility in large demographics. Although, each method offers advantages and contrarily limitations that may need to be overcome, understanding natural infection for a particular pathogen can help guide the optimal delivery route for maximal vaccine response (252).

### 5.4 Clinical Evaluation

Children, older adults and immunocompromised individuals are primary risk groups for MTB, BP and influenza infection. As such, for vaccines designed to reduce transmission of respiratory infection in general, new vaccination strategies should also strive to take into consideration the target population, including for human immune ontogeny and clinical history. For example, demonstrated for influenza (253), exposure to circulating respiratory viruses may imprint a subsequent immune response, with unique T cell responses in early life. Better understanding of the effects of pathogenesis as intertwined with T cell ontogeny is beginning to elucidate more effective vaccine approaches for the induction of early life cell mediated immunity (254–256). Multidisciplinary approaches including standardized human *in vitro* models, systems vaccinology, and innovations of formulation and delivery systems will enhance identification of mechanisms of action and biomarkers of safety and efficacy of adjuvants and adjuvanted vaccines thereby accelerating and de-risking adjuvanted vaccine development (82, 257) (**Figure 2**). Successful stimulation of T cell responses by precision vaccine approaches has certain criteria: i) considering MHC diversity in human population, ii) targeting immunodominant epitopes (either by bioinformatics or by immunophenotyping approaches, iii) assessment method *in vitro* (either tetramer-based assay or targeted antigen stimulation) to evaluate co-relation of protection. There is also a growing interest in the possibility of trained-immunity based vaccines (TIbV), meant to enhance innate cross-protection to heterologous pathogens and to induce more efficient adaptive responses against the specific pathogens contained in the vaccine (258–264).

## 6 Concluding Remarks

Vaccines are one of the most cost-effective and effective interventions to address the global burden of pediatric infectious diseases and the implementation of early life immunizations has reduced deaths in neonates and children across the world (4). Infants are capable of inducing adaptive immune responses after pathogen exposure. Though immune responses may be suboptimal in early age but it could be adequately induced and effectively maintained after vaccination, under certain conditions (265). Due to aging, the elder immune system, especially the innate compartment, gradually wanes *via* the process of immunosenescence, also leaving this population vulnerable to infection (266). Due to such differences in the early life and later life adult immune systems, it is increasingly appreciated that an in-depth understanding of early life immunity is crucial for the development of effective pediatric vaccines and for optimizing geriatric vaccine schedules. The human specific immunopathology leading to protective immunity to MTB, BP and influenza are partially and potentially poorly characterized. Live attenuated vaccines generally provide more robust protective immunity than vaccines comprised of killed organisms. Innate detection of a signatures of microbial viability such as bacterial RNA has been shown to be acted as vita-PAMPs (74). As compared to non-viable pathogens, transient bacterial RNA induces T_FH_ differentiation and enhanced humoral responses (267). Vaccine formulation like BCG or live attenuated BP vaccine strain (BPZE1) or the LAIV, which incorporate molecular signatures of microbial viability, have the potential to establish protective immunity against reinfection that may outperform protein-based vaccines. Furthermore, as immune responses induced by natural infection and pathogen specific vaccines often overlap, an important question remains to be addressed: should natural infection be used as a benchmark for optimal vaccine design, or should the ability of novel vaccines to induce a broad unnatural spectrum of immunity be the goal? Immunological imprinting, after the first influenza infection/s in early life for example, might influence vaccine efficiency by altering the immune response in post vaccination period (268). BCG vaccination imprints a persistent transcriptomic bias on human stem and progenitor cells toward the myeloid cell lineage which elicits beneficial trained immunity (71, 269), an added component of vaccine formulation only elucidated in the most recent decades. Therefore, it may be important to study the potential impact of immune imprinting by natural infections, but to also consider the role of induction of unnatural response to solve some of the most pressing and enduring problems of vaccine preventable and non-preventable diseases. These questions may help guide the vaccine design in 21^st^ century.

## Author Contributions

DD conceived the review article. SB, DS, BB and DD contributed to the writing. EN contributed to intellectual input and critical review. All authors contributed to the article and approved the submitted version.

## Funding

DD’s laboratory is supported by NIH grant 5R21AI137932-02, Adjuvant Discovery Program contract #75N93019C00044, Adjuvant Development Program contract #272201800047C. The *Precision Vaccines Program* is supported in part by the BCH Department of Pediatrics and the Chief Scientific Office.

## Conflict of Interest

DD is a named inventor on granted patents and patent applications related to vaccine adjuvants and vaccine formulation design. DS, BB and EN are named inventor on multiple patent applications focused on the design of the adjuvanted vaccine formulation.

The remaining authors declare that the research was conducted in the absence of any commercial or financial relationships that could be construed as a potential conflict of interest.

## Publisher’s Note

All claims expressed in this article are solely those of the authors and do not necessarily represent those of their affiliated organizations, or those of the publisher, the editors and the reviewers. Any product that may be evaluated in this article, or claim that may be made by its manufacturer, is not guaranteed or endorsed by the publisher.
